# Effect of matrix metalloproteinase inhibitors on microtensile bond strength of dental composite restorations to dentin in use of an etch‐and‐rinse adhesive system

**DOI:** 10.1002/cre2.313

**Published:** 2020-09-29

**Authors:** Aida Saffarpour, Sara Valizadeh, Abolghasem Amini, Mohammad‐Javd Kharazifard, Marzieh Rohaninasab

**Affiliations:** ^1^ Dental Research Center, Dentistry Research Institute Tehran University of Medical Sciences Tehran Iran; ^2^ Department of Operative Dentistry, School of Dentistry Tehran University of Medical Sciences (International Campus) Tehran Iran; ^3^ Restorative Dentistry Department, School of Dentistry Tehran University of Medical Sciences Tehran Iran; ^4^ Tehran University of Medical Sciences Tehran Iran

**Keywords:** etch‐and‐rinse, matrix metalloproteinases, microtensile bond strength

## Abstract

**Aim:**

This study assesses the effect of matrix metalloproteinases on microtensile bond strength (μTBS) of an etch‐and‐rinse adhesive system.

**Methods:**

This in vitro study evaluated 88 extracted premolars. The teeth were sectioned to expose dentin and were then randomly divided into four groups (*n* = 22). In group 1 (control), dentin surface was etched, and Adper Single Bond 2 was applied. In groups 2–4, dentin surface was etched and chlorhexidine (CHX), 1‐ethyl‐3‐(3‐dimethylaminopropyl) carbodiimide (EDC), and dimethyl sulfoxide (DMSO) were applied on the surfaces, respectively, and blotted dry. Next, Adper Single Bond 2 was applied and all teeth were built up with Z350 composite. In each group, half the samples immediately and the other half after 10,000 thermal cycles underwent μTBS test. Data were analyzed using ANOVA and Tukey's test (*α* = .05).

**Results:**

In thermocycled samples, maximum μTBS was noted in CHX group followed by DMSO, EDC, and control group (*p* < .001). The thermocycled μTBS of composite to dentin was significantly higher in CHX group compared with EDC, DMSO, and control groups (*p* < .001) but was not significantly different in EDC and DMSO groups (*p* = .498).

**Conclusion:**

The thermocycled μTBS obtained by the application of CHX, EDC, and DMSO was significantly higher compared with the value to the control group.

## 1 INTRODUCTION

Dental adhesive systems have greatly advanced in the recent years with regard to chemical composition, mechanism of action, technique of application, and clinical efficacy. The adhesive system can bond to dentin via two different strategies. The etch‐and‐rinse adhesive systems eliminate the smear layer while self‐etch adhesives preserve the smear layer as a substrate for bonding (Mallick et al., 2015). The ability of dentin bonding agents to create a hermetic seal and a durable bond to tooth structure is a prerequisite for long‐term service of dental restorations. Formation of a resin‐infiltrated hybrid layer composed of collagen fibers in methacrylate resin results in adhesion. Nonetheless, despite a successful immediate bonding, the durability of the adhesive interface may remain questionable due to the presence of a number of physical and chemical factors compromising the adhesive interface (Tekçe, Tuncer, Demirci, & Balci, 2016).

The durability of adhesive bonding to dentin is influenced by the degradation of resin components through hydrolysis of polymerized hydrophilic resin and destruction of collagen matrix by matrix metalloproteinases (MMPs) and cathepsin cysteine proteases (Breschi et al., 2008).

MMPs and cathepsin are present in the composition of dentin and are believed to be responsible for the hydrolysis of collagen fibers and inhibition of bonding of composite to mineralized dentin in the hybrid layer (Tjaderhane et al., 2013a). Synthetic MMP inhibitors such as quaternary ammonium methacrylate compounds and benzalkonium chloride can be used to increase the durability of resin bonding (Almahdy et al., 2012; Tezvergil‐Mutluay et al., 2011). Other strategies suggested for this purpose include remineralization, ethanol wet bonding, and use of collagen cross‐linkers (Tjaderhane et al., 2013b).

Chlorhexidine (CHX), as a MMP and cathepsin inhibitor, can prevent the degeneration of the bonds to dentin (Breschi et al., 2010; Cecchin, de Almeida, Gomes, Zaia, & Ferraz, 2011; Cecchin et al., 2014; Liu et al., 2011; Scaffa et al., 2012; Shafiei, Doozandeh, & Alavi, 2011). Moreover, evidence supports the positive effects of CHX on the durability of the bonds to dentin. However, these effects have not been definitely confirmed (Davari, Daneshkazemi, Frahat, & Kohestani, 2019; Hussein & Al‐Shamma, 2019). Dissolution in water and the reversible electrostatic bond of CHX can lead to gradual loss of the inhibitory effect of CHX on MMPs after 18–24 months (Ricci, Sanabe, de Souza Costa, Pashley, & Hebling, 2010).

On the other hand, 1‐ethyl‐3‐(3‐dimethylaminopropyl) carbodiimide (EDC) is a stable isomer introduced as a nonspecific cross‐linker protein with low toxicity (Scheffel et al., 2015). It can inactivate the catalytic and cathepsin parts of MMPs by the activation of carboxylic groups and amino‐acid cross‐links. Recently, the positive effects of EDC on the durability of the bonds of etch‐and‐rinse adhesives were reported (Mazzoni et al., 2014; Tezvergil‐Mutluay et al., 2012).

Dimethyl sulfoxide (DMSO) is a bipolar protic solvent (a protic solvent is a solvent that has a hydrogen atom bound to an oxygen atom) that enhances the penetration depth of resin into biological surfaces. DMSO molecule has a S=O polar group and two hydrophobic CH3 groups. DMSO is completely dissolved in all solvents such as resin monomers of the adhesive systems (Marren, 2011) and can separate the collagen network and modify the inter‐fibrillar spaces in the dentin matrix (Yeh, Choi, Nelson, & Tromberg, 2003; Zimmerley, McClure, Choi, & Potma, 2009), which can lead to suppression of intra‐peptide hydrogen bonds in the collagen matrix (Vishnyakov, Lyubartsev, & Laaksonen, 2001). Researchers recently found that DMSO can improve and preserve the adhesive bonding for long term. Thus, it can be used to promote adhesive bonding by inhibiting MMPs (Carrilho et al., 2007; Pashley et al., 2011).

**FIGURE 1:**
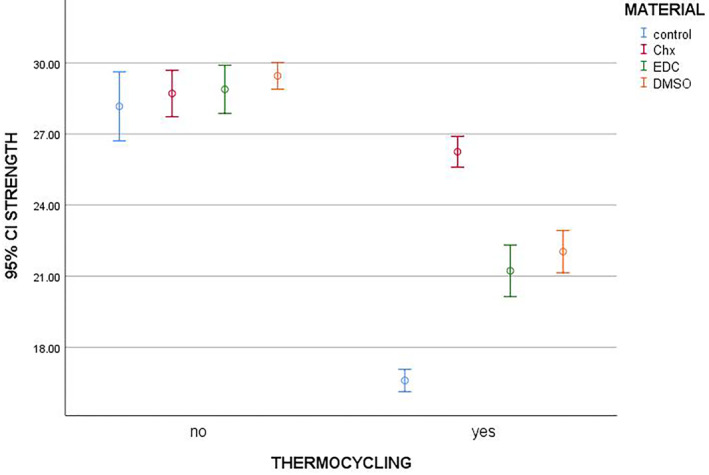
Mean and *SD* of microtensile bond strength (μTBS) of composite to dentin with and without thermocycling

The positive effects of CHX on the durability of the bonds created by etch‐and‐rinse adhesives have been previously reported (Breschi et al., 2010; Cecchin et al., 2011, 2014; Liu et al., 2011; Scaffa et al., 2012; Shafiei et al., 2011). Some studies have evaluated the inhibitory effects of DMSO on MMPs and its positive effects on the durability of the bond of fiber posts (Scaffa et al., 2012). The protective effects of EDC on the collagen structure and durability of the bond of fiber posts and its inhibitory effects on MMPs have been previously investigated (Breschi et al., 2010; Cecchin et al., 2011, 2014; Davari et al., 2019; Hussein & Al‐Shamma, 2019; Liu et al., 2011; Ricci et al., 2010; Scaffa et al., 2012; Scheffel et al., 2015; Shafiei et al., 2011). However, no previous study has compared CHX, DMSO, and EDC in this respect. Thus, this study aimed to assess and compare the effects of these three MMP inhibitors on microtensile bond strength (μTBS) of dental restorations immediately and after thermocycling in use of an etch‐and‐rinse adhesive system. Our null hypothesis was that different MMP inhibitors would not affect the bond strength of resin composite to dentin after thermocycling (Figure 1).

## 2 MATERIALS AND METHODS

This in vitro, experimental study was approved by the ethics committee of Tehran University of Medical Sciences (1396.2810.IR.TUMS.DENTISTRY.REC). A total of 88 extracted sound premolars with no cracks or caries that had been extracted as part of orthodontic treatment (due to space shortage) were used in this study after obtaining the patients' consent. Teeth with cracks, fractures, carious lesions, defects, or anomalies were excluded. After extraction, debris and soft tissue residues were removed and the teeth were immersed in 0.5% chloramine T solution at 4°C for 1 week. They were then stored in saline and incubated at 37° for 1 month.

Dentin surface was then exposed using a 1‐mm diamond bur (#878d2, Teezkavan, Tehran, Iran) perpendicular to the occlusal surface under air and water spray. Samples were mounted and fixed with isomet to standardize the procedure during occlusal enamel removal. The bur was replaced after preparation of five teeth. The surface of the teeth was then polished using Silicone carbide paper grades of P600 under copious water irrigation for 30 s in order to create a smear layer. The teeth were then randomly divided into four groups (*n* = 22).

Group 1 (control): The teeth were etched and then Adper Single Bond 2 was applied on their surface according to the manufacturer's instructions (Table 1).
Group 2: After etching, 0.5 mL of 0.02% CHX was applied on dentin surface for 60 s and blotted dry.
Group 3: After etching, 1 mL of 0.3 M EDC was applied on dentin surface and blotted dry.
Group 4: After etching, 1 mL of 5% aqueous solution of DMSO was applied on the surface of the teeth and blotted dry.

**TABLE 1:** Materials used in this study

Material	Composition	Manufacturer	Application technique
Adper Single Bond 2	Ethyl alcohol, Bis‐GMA, HEMA, glycerol, dimethacrylate, acrylic acid copolymer, Itaconic acid, diurethane dimethacrylate, water, colloidal filler	3M Oral Care, St. Paul, MN	37% phosphoric acid gel was used to etch dentin surfaces for 15 s. The etched dentin surfaces were then rinsed for 10 s to completely remove the etching gel. Then, the adhesive was applied on the wet dentin with a microbrush and rubbed for 20 s followed by gentle air drying for 5 s, and the second layer was applied and gently air‐dried and light‐cured for 20 s using Demi Ultra (Kerr, Brea, CA) light‐curing unit
Filtek Z350 composite	Bis‐GMA, UDMA, TEGDMA, ethyl methacrylate; inorganic fillers	3M Oral Care Dental Products, St. Paul, MN	In all groups, A2 shade of Z350 composite (3 M Oral care) was applied on the surface in one 1‐mm and two 1.5‐mm thick increments and each layer was light‐cured for 20 s using Demi Ultra (Kerr, Brea, CA) light‐curing unit
Scotchbond etchant	37% phosphoric acid	3M Oral Care Dental Products, St. Paul, MN	Dentin surface was conditioned with 37% phosphoric acid for 15 s and then rinsed with water spray. Excess water was removed by an applicator to leave a moist, but not wet, surface (blot‐dry technique)
CHX	0.02% chlorohexidine	Ultradents Pruducts, Inc, South Jordan, UT	
EDC	0.3 Mol 1‐ethyl‐3‐(3‐dimethylaminoprophyl) carbodiimide		
DMSO	5% Aquas solution Dimethylsulfuxide		

Next, the bonding procedure was continued using a two‐step etch‐and‐rinse bonding agent (Adper Single Bond 2). The matrix band is placed around each tooth after bonding application to make composite insertion and doing restoration easier. Then, the teeth were built up using Z350 composite (3 M Oral Care; Table 1). All procedures were performed according to the manufacturers' instructions. The matrix band was then removed and the teeth were finished and polished.

In all four groups, half of the samples (*n* = 44) were prepared for μTBS test in a universal testing machine (Zwick Roell, Germany). The teeth were then sectioned into 1‐mm thick sections, turned 90°, and sectioned again to obtain dentin–resin sections with triangular cross‐sections measuring 1 mm^2^ using Accutom 50 sectioning machine (Struers, Copenhagen, Denmark). Three to four sections were made in each restoration. The samples were placed in the clamp of universal testing machine and tensile load was applied perpendicular to the bonding interface. The tensile load that caused failure at the interface was recorded. The remaining half of the samples were subjected to 10,000 thermal cycles in a thermocycler between 5 and 55°C (Gale & Darvell, 1999). The dwell time was 20 s and the transfer time was 10 s. Next, they underwent μTBS test at a crosshead speed of 0.5 mm/min.

Data were analyzed using SPSS version 16 via ANOVA and Tukey's test. Level of significance was set at .05.

## 3 RESULTS

According to two‐way ANOVA, the effect of thermocycling and type of MMP inhibitor on immediate and post thermocycling μTBS of composite to tooth structure was significant (*p* < .001). The interaction effect of thermocycling and type of MMP inhibitor on immediate and post thermocycling μTBS of composite to dentin was also significant (*p* < .001).

Maximum immediate μTBS was noted in DMSO followed by EDC, CHX, and control groups. According to ANOVA, in samples that did not undergo thermocycling, the immediate μTBS of composite to tooth structure was not significantly different in the control, CHX, EDC, and DMSO groups (*p* = .319, Table 2).

**TABLE 2:** Comparison of μTBS of composite to dentin in the groups

Thermocycling	Groups	Mean	*SD*	Minimum	Maximum	*p‐*value
No	Control^a^	28.165	3.901	15.06	32.63	.319
	CHX^a^	28.709	2.984	18.87	33.14	
	EDC^a^	28.883	2.775	18.06	32.67	
	DMSO^a^	29.454	1.759	21.12	33.03	
Yes	Control^b^	16.593	1.291	12.06	18.03	<.001
	CHX^c^	26.248	1.868	19.93	28.73	
	EDC^d^	21.221	3.260	12.36	33.17	
	DMSO^d^	22.032	2.428	12.06	24.83	

Abbreviations: CHX, chlorhexidine; DMSO, dimethyl sulfoxide; EDC, 1‐ethyl‐3‐(3‐dimethylaminopropyl) carbodiimide; μTBS, microtensile bond strength.

Among the thermocycled samples, maximum post thermocycling μTBS was noted in CHX group followed by DMSO, EDC, and control groups. According to ANOVA, among the thermocycled samples, a significant difference was noted in post thermocycling μTBS of composite to tooth structure in the control, CHX, EDC, and DMSO groups (*p* < .001, Table 2).

The Tukey's test showed that in thermocycled samples, post thermocycling μTBS of composite to tooth structure in the control group was significantly lower than that in all other groups (*p* < .001). The μTBS of composite to tooth structure in CHX group was significantly higher than that in EDC, DMSO, and control groups (*p* < .001). The μTBS of composite to tooth structure was not significantly different between EDC and DMSO groups (*p* = .498).

## 4 DISCUSSION

At present, the main challenge in use of dental adhesive systems is related to the factors affecting the bond strength to dentin (Mazzoni et al., 2015). The quality of collagen fibers depends on the stability of dentin hybrid layer; spontaneous destruction of collagen fibers in this layer occurs following the application of dentin bonding agents due to the gradual effect of MMPs (Ghavam et al., 2009). Recently, use of MMP inhibitors was recommended aiming to enhance the durability of dentin bonding and prevention of dentin demineralization at the interface (Frassetto et al., 2016).

Search of the literature by the authors yielded no previous study comparing the efficacy of CHX, DMSO, and EDC MMP inhibitors. Thus, this study compared the inhibitory effects of MMPs on μTBS of dental restorations in the use of an etch‐and‐rinse adhesive system. Several macro and micro tests are available for assessment of bond strength. In the present study, the μTBS test was used due to advantages such as preparation of several samples from each tooth, better control of differences and better stress distribution at the actual interface (Sirisha, Rambabu, Ravishankar, & Ravikumar, 2014). Many studies have reported that thermocycling decreases the bond strength to enamel and dentin. Thus, the samples underwent 10,000 thermal cycles in our study to simulate 1 year of clinical service of restorations (Koshy, n.d.).

In this study, etch‐and‐rinse adhesive was used to assess the effect of MMP inhibitors on the collagen network because the acidic environment created by acid etching of dentin causes further stimulation of MMPs in the peripheral dentin layer. Activation of MMPs and cathepsins during the bonding process with the use of etch‐and‐rinse adhesives causes degradation of coronal or radicular dentin collagen. Activation of MMPs and cathepsins may occur as the result of low primary pH of volatile residual acidic monomers (Serino et al., 2019).

Most studies use 2% concentration of CHX for inhibition of MMPs (Vallabhdas et al., 2018). However, Breschi et al. (2010) and Mazzoni et al. (2011) found that CHX can inhibit MMPs even in 0.2% concentration. Thus, 0.2% CHX was used in this study. CHX effectively inhibits MMP2, MMP8, MMP9, and cysteine cathepsin proteases (Mazzoni et al., 2011).

The current results showed that in samples that did not undergo thermocycling, the immediate μTBS of composite to tooth structure in CHX, EDC, and DMSO groups was not significantly different from that in the control group (*p* = .319). Similarly, Chang et al. used CHX prior to the application of adhesive on dentin and found no significant difference in immediate μTBS (Chang & Shin, 2010). Tjaderhane et al. (2013b) applied DMSO and found no significant difference in immediate μTBS. Thus, it seems that the effects of MMP inhibitors cannot be witnessed in short‐term.

The current results revealed that the long‐term μTBS of composite to tooth structure in CHX group was significantly higher than that in the control group (*p* < .001). This finding can be attributed to the activity of MMPs and degradation of collagen layer in the control group (Almahdy et al., 2012; Strobel & hellwig, 2015). The obtained results are similar to those of previous studies (Mazzoni et al., 2014; Tezvergil‐Mutluay et al., 2012). In this study, the bond strength of CHX group was significantly higher than that of the control group. This finding was in agreement with the results of in vitro and in vivo studies that used Single Bond adhesive. Similarly, Zheng et al. reported maximum bond strength in CHX group. They demonstrated that application of CHX further stabilized the collagen fibers after 24 hr and 3 months and yielded higher protection. It seemed that maximum penetration of adhesive into the collagen network occurred in the CHX group and resulted in higher adhesive bond strength in this group. This also increased the resin–dentin bond strength. Moreover, CHX showed maximum micro‐penetration (Zheng & Chen, 2017). Similarly, Chang et al. reported that the effects of thermocycling and CHX and the interaction effect of the two on long‐term μTBS of composite to tooth structure were significant (Chang & Shin, 2010).

In the present study, the μTBS of EDC and DMSO groups after 10,000 thermal cycles was significantly higher than that in the control group. In line with our findings, Shafiei, Memarpour, and Sarafraz (2016) reported that the application of DMSO significantly increased the bond strength of adhesive‐cemented fiber posts in the root canal system after aging. Also, Shafiei, Yousefipour, and Mohammadi‐Bassir (2016) reported that application of EDC significantly increased the bond strength of adhesive‐cemented fiber posts in the root canal system after aging. However, the results of the abovementioned two studies cannot be compared with our findings due to differences in methodologies.

Tjaderhane et al. (2013b) reported that the application of DMSO significantly increased the long‐term μTBS after 6 and 12 months, compared with the control group. Recently, it was reported that 5% DMSO can inactivate MMP‐2 and MMP‐9 human gelatinases. When used as dentin primer, DMSO can prevent the degradation of resin‐dentin bonding interface after 12 months of aging (Chang & Shin, 2010; Osorio et al., 2011). It seems that the application of DMSO may increase the wetting of collagen and enhance the penetration of adhesive between the collagen fibers exposed by acid etching. Thus, resin impregnation of demineralized dentin can be improved as such (Shafiei, Memarpour, & Sarafraz, 2016).

The positive effects of EDC on the durability of the bond can be related to its inhibitory effects on MMPs. Evidence shows that EDC can deactivate the catalyst sites of MMPs and cathepsins. This is done by the activation of carboxyl groups and cross‐linking of amino‐acids, and subsequent formation of new, stable covalent peptide bonds, and decreased molecular mobility (Shafiei, Yousefipour, & Mohammadi‐Bassir, 2016).

In the present study, no significant difference was noted between EDC and DMSO groups (*p* = .498) but the μTBS of the CHX group was significantly higher than that of EDC and DMSO groups (*p* < .001). It seems that despite the positive effects of DMSO and EDC on bond strength, CHX can better protect the bonding interface over time.

A successful MMP inhibitor has effective functional groups namely the carboxylic and hydroxamic groups. These functional groups have the potential to chelate the active sites of MMP molecules. Also, MMP inhibitors in combination with dentin adhesives may enhance the interlocking reaction between substrates and inhibitors. Combination of MMP inhibitors and dental adhesive systems can increase the micromechanical interlocking via reactions between MMP substrate and inhibitors (Frassetto et al., 2016).

Based on the current results, CHX, DMSO, and EDC are suitable materials for providing higher bond strength value over time; however, considering the superior results of CHX, its lower cost, easier availability, and simpler application, as well as its immediate antibacterial activity, it seems that the application of CHX is more efficient for the preservation of the hybrid layer. Clinical trials are required to assess the effect of MMP inhibitors on resin bond strength to dentin. Furthermore, the assessment of bond strength is recommended by the use of scanning electron microscopy.

## 5 CONCLUSION

The current results revealed that in vitro application of CHX, EDC, and DMSO had no significant effect on the immediate μTBS of composite to tooth structure without thermocycling. After thermocycling, μTBS decreased in all groups but maximum post thermocycling μTBS of composite to tooth structure was noted in CHX group significantly higher than other groups, followed by DMSO and EDC groups, which were significantly higher than control group with no significant difference with each other.
